# Supplementary material for: Structure of the hyperosmolality-gated calcium-permeable channel OSCA1.2
Source: Nat Commun. 2018 Nov 29;9:5060. doi: 10.1038/s41467-018-07564-5 (PMC6265326; doi:10.1038/s41467-018-07564-5)
Supplement: Supplementary file 1 — Supplementary Information [file 41467_2018_7564_MOESM1_ESM.pdf]

Supplementary Information

Structure of the hyperosmolality-gated calcium-permeable channel OSCA1.2

Liu et al.

Supplementary Figures and Figure Legends

a

Sequence Identities (upper triangle) / Sequence Similarities (lower triangle)

	OSCA1.1	CSC1/ OSCA1.2	OSCA1.3	OSCA1.4	OSCA1.5	OSCA1.6	OSCA1.7	OSCA1.8	OSCA2.1	OSCA2.2	OSCA2.3	OSCA2.4	OSCA2.5	OSCA3.1	OSCA4.1
OSCA1.1		85.3	72.7	71.1	71.8	66.8	61.8	57.5	29.1	27.0	32.3	28.7	29.7	28.3	15.3
CSC1/ OSCA1.2	92		73.5	73.7	71.7	66.9	62.1	58.1	29.5	28.7	33.8	30.9	29.8	28.5	15.8
OSCA1.3	84	86		82.4	68.1	65.6	62.8	57.3	28.6	27.2	32.3	28.2	30.6	29.2	15.7
OSCA1.4	83	84	89		67.6	65.9	61.0	55.9	28.4	26.0	32.4	28.6	29.3	27.5	14.4
OSCA1.5	83	85	82	82		78.0	64.8	58.9	29.4	27.9	32.6	30.0	29.4	28.4	15.4
OSCA1.6	82	82	80	81	88		60.8	55.3	28.8	27.9	31.1	28.9	28.6	27.7	14.7
OSCA1.7	77	77	78	78	81	78		56.8	28.6	27.8	32.3	29.5	28.4	29.5	14.7
OSCA1.8	77	77	78	76	79	77	74		29.3	28.5	31.8	30.2	29.3	30.9	15.1
OSCA2.1	49	52	55	54	57	54	54	56		77.0	42.9	40.2	35.8	24.8	14.2
OSCA2.2	52	54	52	52	52	53	52	54	87		42.2	39.4	35.1	23.7	14.7
OSCA2.3	54	53	55	50	52	50	50	54	62	63		52.5	37.9	23.0	16.6
OSCA2.4	52	52	52	54	52	53	51	52	59	60	70		34.6	23.1	15.2
OSCA2.5	53	53	51	49	51	52	52	53	54	53	56	52		24.4	14.1
OSCA3.1	52	51	50	50	51	51	52	51	46	47	46	47	46		15.1
OSCA4.1	40	40	41	40	39	39	37	40	38	38	39	38	41	36	

b

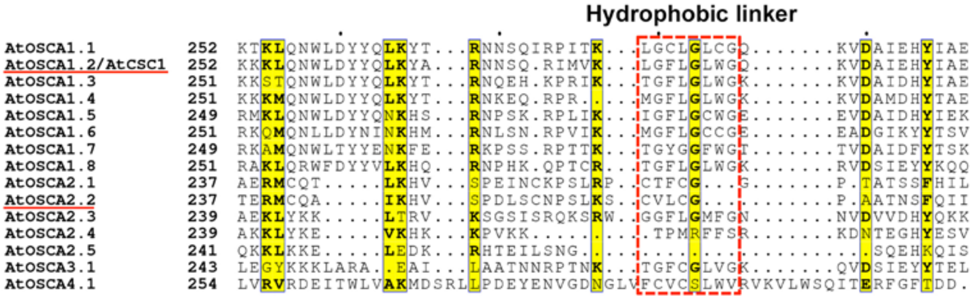

Supplementary Figure 1. Sequence alignment of AtOSCA family proteins

a Sequence conservation between AtOSCA family proteins. Pair-wise sequence comparison was performed with online services of Blastp in NCBI (National Center for Biotechnology Information). **b** This linker is highly conserved in AtOSCA proteins, except in AtOSCA2.1-2.2 or AtOSCA2.4-2.5. Sequence alignment is presented using ESPript[1].

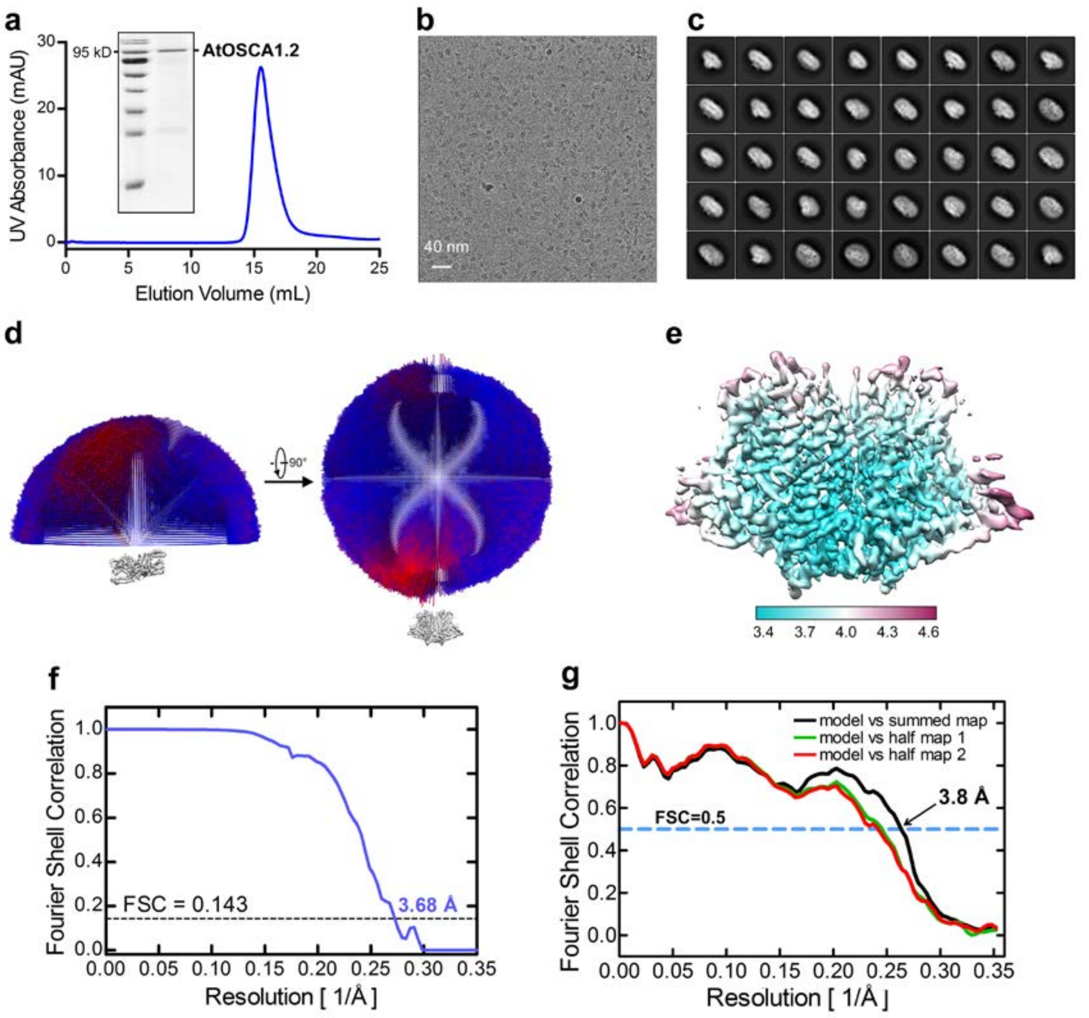

Supplementary Figure 2. Cryo-EM analysis of AtOSCA1.2

a A representative gel filtration analysis of AtOSCA1.2 in digitonin. The mono-disperse peak suggests an excellent solution behavior of the sample. The peak fraction from a Sepharose-6 column (10/30, GE healthcare) was visualized on SDS-PAGE by Coomassie blue staining. Source data are provided as a Source Data file. **b** A representative cryo-EM micrograph. **c** Representative reference-free 2D class averages of the AtOSCA1.2 particles. **d** Euler angle distribution of the final 3D refinement of overall map. **e** Local resolution maps calculated using ResMap. **f** The gold-standard Fourier shell correlation curves for the overall map. **g** FSC curves of the refined model versus the overall 3.68 Å map that it was refined against (black); of the

model refined in the first of the two independent maps used for the gold-standard FSC versus that same map (green); and of the model refined in the first of the two independent maps versus the second independent map (red). The small difference between the red and green curves indicates that the refinement of the atomic coordinates did not suffer from overfitting.

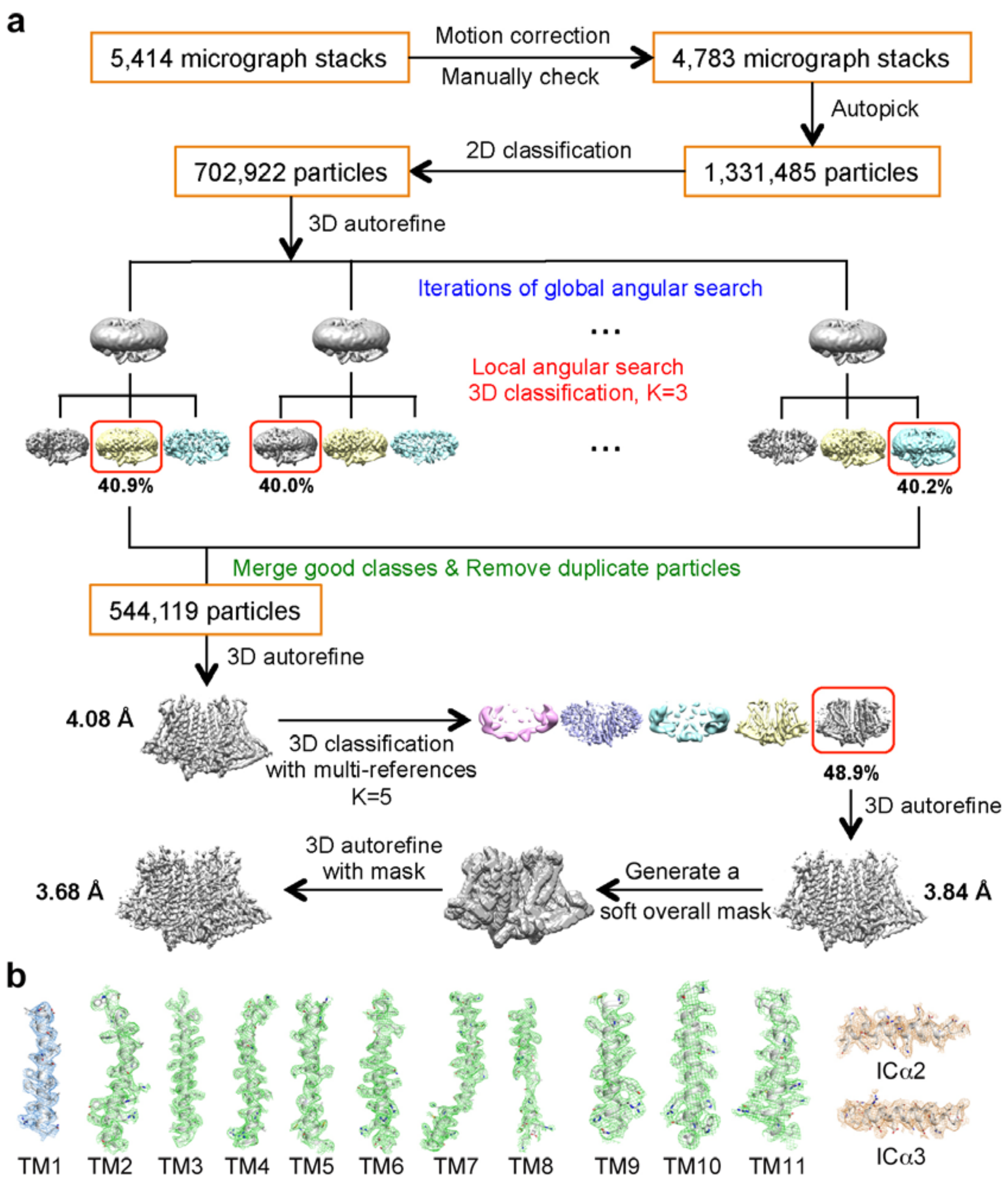

Supplementary Figure 3. Cryo-EM data processing

a A flowchart for cryo-EM data processing. Details can be found in the “Image processing” session in Methods. **b** Representative EM densities for AtOSCA1.2. EM density map and the atomic model are shown for all 11 TMs and the two long cytosolic helices of AtOSCA1.2.

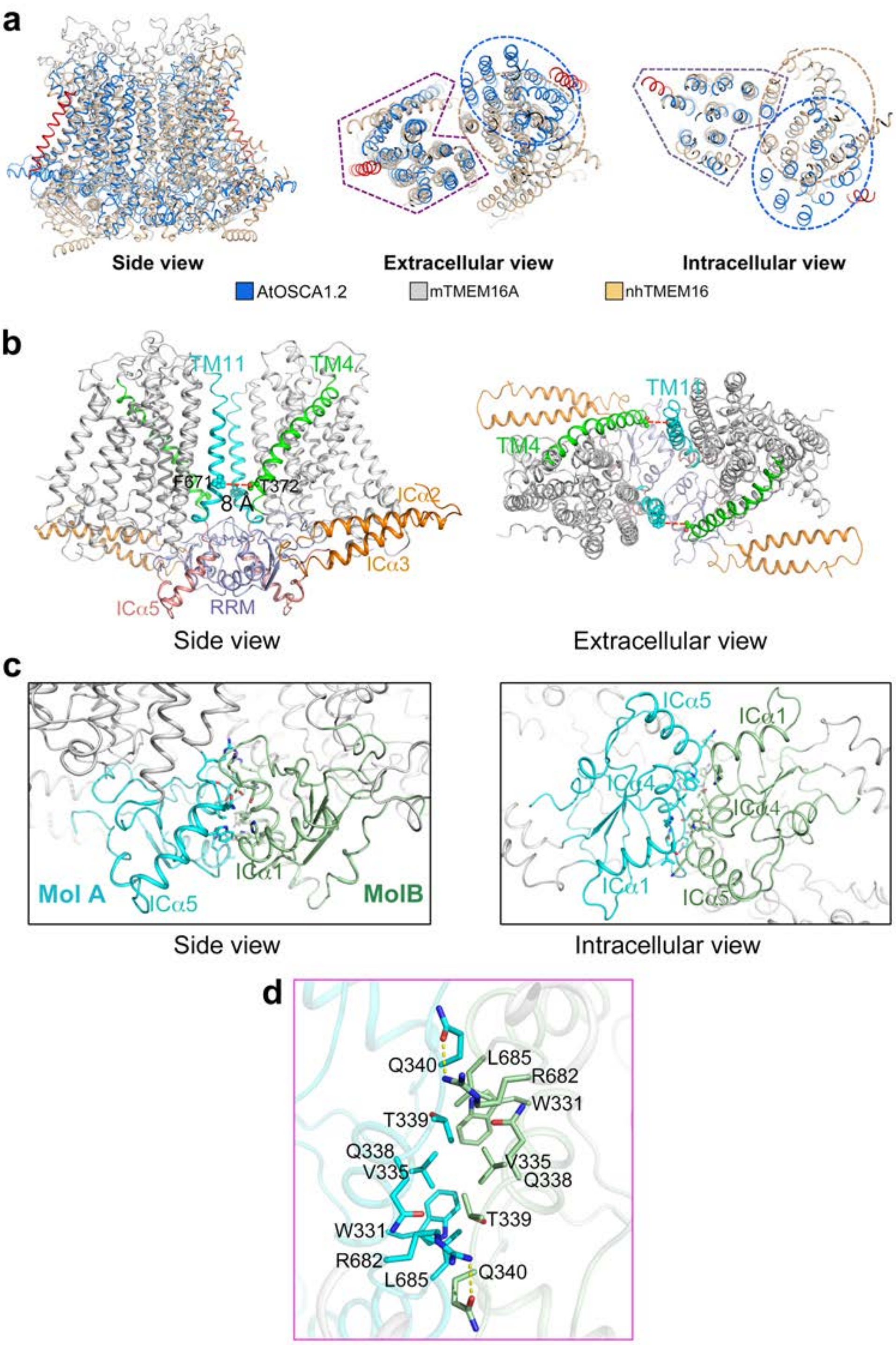

Supplementary Figure 4. Structure comparison between AtOSCA1.2, mTMEM16A and nhTMEM16

a The dimer organization of AtOSCA1.2 is different from mTMEM16A or nhTMEM16. Structural alignments for the dimeric AtOSCA1.2, mTMEM16A (PDB code: 5OYB [http://dx.doi.org/10.2210/pdb5OYB/pdb]) and nhTMEM16 (PDB code:

4WIT [http://dx.doi.org/10.2210/pdb4WIT/pdb]) are shown in three views. While one monomer of AtOSCA1.2 aligns well to mTMEM16A or nhTMEM16, the other monomer has a large movement. The transmembrane helices of AtOSCA1.2, mTMEM16A or nhTMEM16 are profiled by dash lines. **b** The transmembrane region of AtOSCA1.2 is not involved in dimer formation. The closest distance between adjacent TMs of two monomers, TM4 of one monomer and TM11 of another, is about 8 Å, as indicated. **c** The cytosolic domain mediates the dimer formation of AtOSCA1.2. Two perpendicular views are shown. ICα1, ICα4 of the RRM and ICα5 are involved in the interactions. **d** Residues on the dimeric interface. Q340 and R682 form an inter-chain hydrogen bond. W331, V335 and L685 form intense hydrophobic interactions.

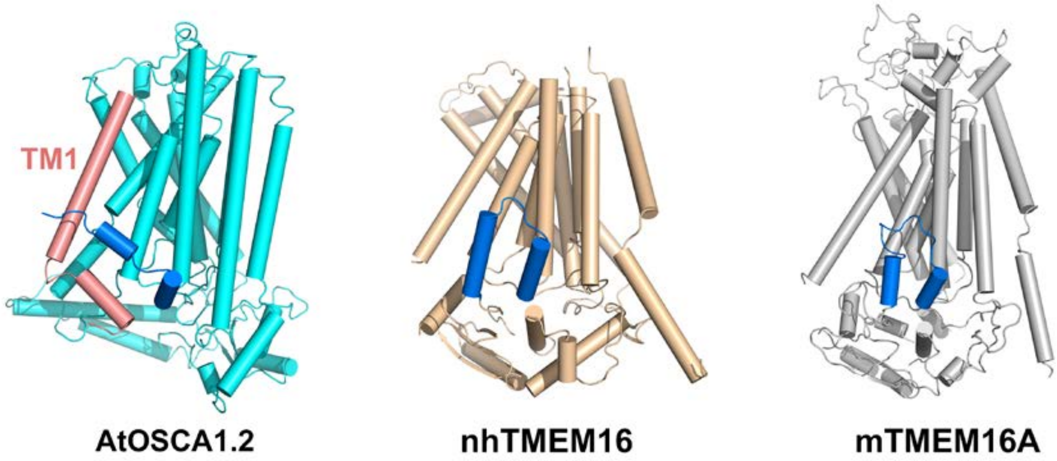

Supplementary Figure 5. Short-helical structures in AtOSCA1.2

Two short α helices prior to TM1 in nhTMEM16 and mTMEM16A form a hairpin structure, but are pulled apart and almost perpendicular to each other in AtOSCA1.2. TM1 and the following short helix of AtOSCA1.2 are colored red. The hairpin helices are colored blue. PDB codes for nhTMEM16 and mTMEM16A are 4WIT [http://dx.doi.org/10.2210/pdb4WIT/pdb] and 5OYB [http://dx.doi.org/10.2210/pdb5OYB/pdb], respectively.

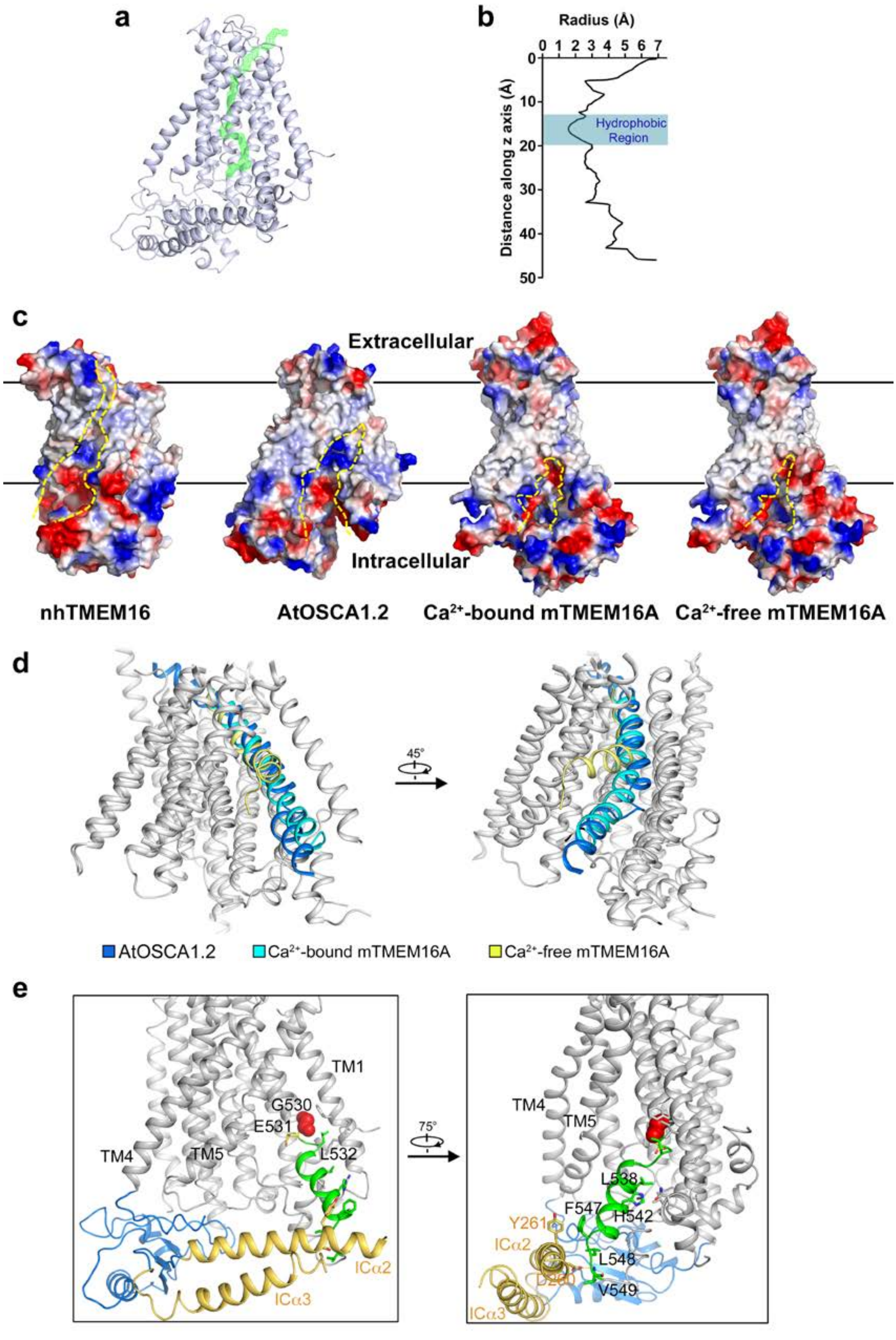

Supplementary Figure 6. Ion conduction pore of AtOSCA1.2

a The ion conduction pore, calculated using HOLE[2], is shown by green mesh. **b** The

pore radius along the channel. The narrowest part is constrained by the hydrophobic residues along the channel. **c** The groove size varies in nhTMEM16, AtOSCA1.2 and mTMEM16A. In nhTMEM16, a hydrophilic membrane-traversing groove is identified to be the catalytic site for lipid scrambling (PDB code: 4WIT [http://dx.doi.org/10.2210/pdb4WIT/pdb]). In AtOSCA1.2, a groove which extends nearly halfway into the membrane from the intracellular side is observed, much larger than that observed in Ca^{2+}-bound (PDB code: 5OYB [http://dx.doi.org/10.2210/pdb5OYB/pdb]) or Ca^{2+}-free (PDB code: 5OYG [http://dx.doi.org/10.2210/pdb5OYG/pdb]) mTMEM16A structures. Shape of the groove is indicated by the yellow dash line. **d** TM7 of AtOSCA1.2 resembles the Ca^{2+}-bound, activated conformation observed in mTMEM16A. Two side views are shown here. TM7 of AtOSCA1.2, TM6 of the Ca^{2+}-bound and Ca^{2+}-free mTMEM16A are colored blue, cyan and yellow, respectively. **e** The carboxyl-terminus of TM7 interacts with ICα2. The intracellular part of TM7 (colored green) rests in a state that resembles the Ca^{2+}-bound structure of mTMEM16A. Its carboxyl-terminus is in close contact with ICα2 through F547, L548 and V549 (colored orange). A hydrogen bond is found in the structure model formed by D260 and V549.

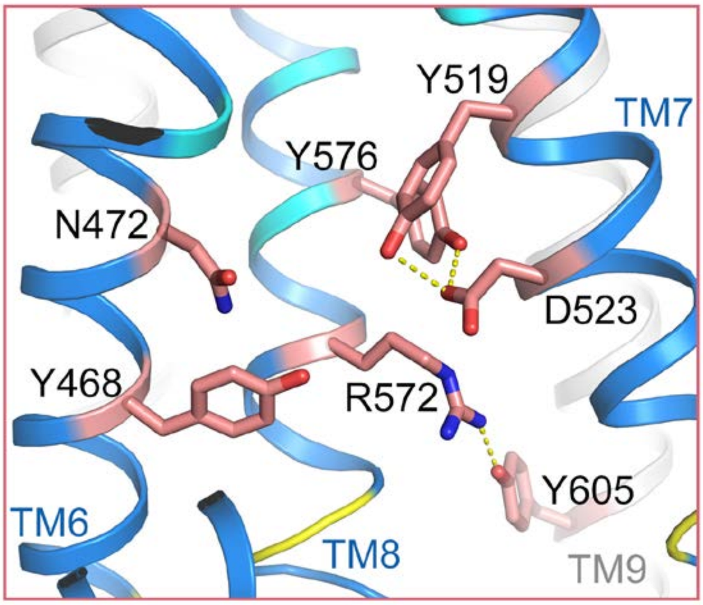

Supplementary Figure 7. Polar and charged residues under the hydrophobic-constricting region of the ion conduction pore

D523 on TM7 forms hydrogen bonds with Y519 on TM7 and Y576 on TM8, respectively. R572 forms a hydrogen bond with Y605 on TM9.

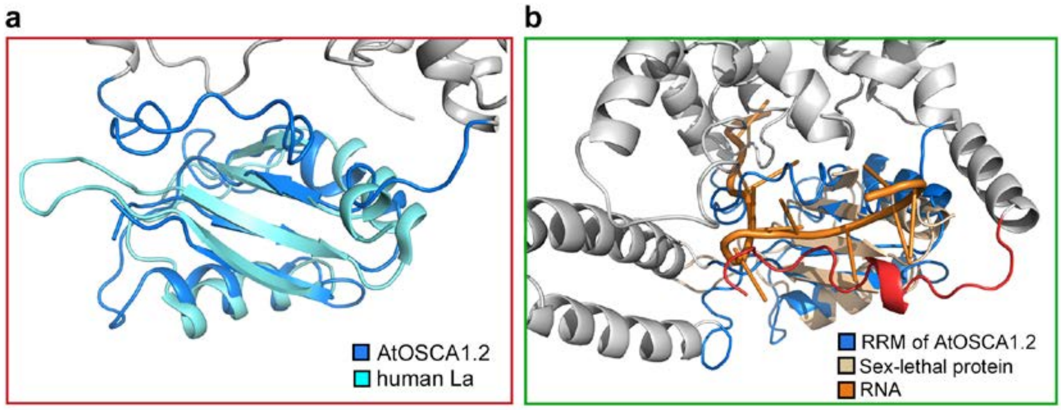

Supplementary Figure 8. RRM motif of AtOSCA1.2

a Structural alignment of the RRM of AtOSCA1.2 (colored blue) with human La protein (colored cyan, PDB code:1S79 [http://dx.doi.org/10.2210/pdb1S79/pdb]). Human La protein is a conserved component of eukaryotic ribonucleoprotein complex that binds RNA through its RRM. **b** Structural comparison between the RRM of AtOSCA1.2 with a RNA/protein complex. Shown here is the sex-lethal protein of *Drosophila melanogaster* in complex with the RNA molecule (PDB code: 1B7F [http://dx.doi.org/10.2210/pdb1B7F/pdb]). The RRM of AtOSCA1.2 is colored blue and the RNA molecule is colored orange. The canonical β-sheet surface for RNA binding is partly blocked by the loop prior to TM4 and the carboxyl-terminus of AtOSCA1.2 (colored red) in current structure.

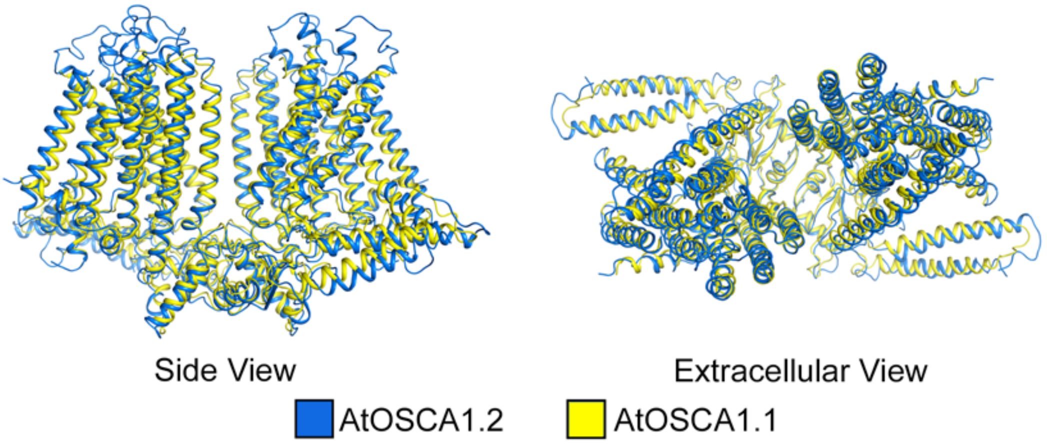

Supplementary Figure 9. Structure alignments between AtOSCA1.1 and AtOSCA1.2

The overall architectures are quite similar and the root mean square deviation between AtOSCA1.2 (colored blue) and AtOSCA1.1 (colored yellow, PDB code: 5YD1 [http://dx.doi.org/10.2210/pdb5YD1/pdb]) is 1.6 Å. Differences are observed in the pore-forming helices like TM4-7 and the two long cytosolic helices.

Supplementary Tables

Primer Name	Sequence
AtOSCA1.2-5'	5'-GATCCCATATGATGGCGACACTTCAGGATATTG-3'
AtOSCA1.2-3'	5'-GATGCTCGAGGACTAGTTTACCACTAAAG-3'
AtOSCA2.2-5'	5'-GATCCCATATGATGGATGTCTCAGCACTTTTAAC-3'
AtOSCA2.2-3'	5'-GATGCTCGAGTGTTCGTTTGTAGATCTC-3'

Supplementary Table 1. Primers used in this study for the subcloning of OSCA1.2 and OSCA2.2 in *Arabidopsis thaliana*.

	AtOSCA1.2 (EMDB-9682) (PDB 6IJZ)	AtOSCA2.2 (EMDB-9677)
**Data collection and processing**		
Magnification	22,500	22,500
Voltage (kV)	300	300
Electron exposure (e–/Å^2)	50	60
Defocus range (μm)	-1.6~-2.5	-1.6~-2.5
Pixel size (Å)	1.31	1.04
Symmetry imposed	C2	C2
Initial particle images (no.)	1,331,485	483,888
Final particle images (no.)	271,802	123,777
Map resolution (Å)	3.68	5.4
FSC threshold	1.43	1.43
Map resolution range (Å)	2.61~999	2.61~999
**Refinement**		
Initial model used (PDB code)	Ab initio model	
Model resolution (Å)	3.68	
FSC threshold	1.43	
Model resolution range (Å)	2.61~999	
Map sharpening *B* factor (Å^2)	-224	
Model composition		
Non-hydrogen atoms	10,964	
Protein residues	1,366	
Ligands	0	
R.m.s. deviations		
Bond lengths (Å)	0.008	
Bond angles (°)	1.100	
Validation		
MolProbity score	1.77	
Clashscore	4.03	
Poor rotamers (%)	0.52	
Ramachandran plot		
Favored (%)	88.77	
Allowed (%)	10.93	
Disallowed (%)	0.3	

Supplementary Table 2. Statistics of Cryo-EM data collection, 3D reconstructions and model refinement of AtOSCA1.2.

Supplementary References

1 Robert, X. & Gouet, P. Deciphering key features in protein structures with the new ENDscript server. *Nucleic Acids Res* **42**, W320-324 (2014).

2 Smart, O. S., Neduvelil, J. G., Wang, X., Wallace, B. A. & Sansom, M. S. HOLE: a program for the analysis of the pore dimensions of ion channel structural models. *J Mol Graph* **14**, 354-360, 376 (1996).
