# Supplementary material for: Structure of the hyperosmolality-gated calcium-permeable channel OSCA1.2
Source: Nat Commun. 2018 Nov 29;9:5060. doi: 10.1038/s41467-018-07564-5 (PMC6265326; doi:10.1038/s41467-018-07564-5)
Supplement: Supplementary file 3 — Description of Additional Supplementary Information [file 41467_2018_7564_MOESM3_ESM.docx]

**Title:** Source Data

**Description:** Contains the original gel image underlying Supplementary Figure 2a
